# Complete Genome Sequence of *Escherichia* Phage Lw1, a New Member of the RB43 Group of Pseudo T-Even Bacteriophages

**DOI:** 10.1128/genomeA.00743-13

**Published:** 2013-12-12

**Authors:** Alla I. Kushkina, Fedor I. Tovkach, André M. Comeau, Igor E. Kostetskii, Igor Lisovski, Andriy M. Ostapchuk, Sergiy I. Voychuk, Tetiana I. Gorb, Liudmyla V. Romaniuk

**Affiliations:** D.K. Zabolotny Institute of Microbiology and Virology of the National Academy of Sciences of Ukraine, Kyiv, Ukrainea; Institut de Biologie Intégrative et des Systèmes, Université Laval, Quebec, Quebec, Canadab; CJSC “Indar,” Protein Engineering Lab, Kyiv, Ukrainec

## Abstract

RB43-related bacteriophages have a specific genome type that clearly distinguishes them from other T4-like viruses. Here, we present the complete genome sequence of a new virulent phage, Lw1, isolated as an *Escherichia coli* BL21(DE3) contaminant. Lw1 shares an RB43-like genome organization, but it does not contain putative AP2-domain endonuclease genes.

## GENOME ANNOUNCEMENT

At the moment, the taxonomy of T4-like bacteriophages is questionable and does not reflect existing relationships between the viruses belonging to this group. Clustered by the morphological principle, the phages often appear to be phylogenetically distant from their prototype T4. Therefore, the isolation and sequencing of additional members of already-existing subgroups of T4-like phages are essential for improvement of the current classifications. RB43-related phages feature a specific genome subtype that differentiates them from other known T4-like phages (1). Currently, this group includes the enterobacterial phages RB16 (1) and RB43 (2), *Klebsiella* phages KP15 and KP27 (3) (GenBank accession no. NC_014036 and HQ918180, respectively), and *Cronobacter* phage vB_CsaM_GAP161 (4). All these phages carry from 1 to 6 putative HNH endonuclease genes coding for AP2-family domains, originally thought to be plant specific (5). Here, we report the annotation of the complete genome sequence of phage Lw1, which shares an RB43-like genome arrangement but does not contain putative AP2-domain genes.

Lw1 was isolated as a contaminating phage from lysed cells of *Escherichia coli* BL21(DE3). Electron microscopic analysis of the phage samples stained with uranyl acetate (2%) showed that Lw1 belongs to the *Myoviridae* family, morphotype A2 (6). The average phage Lw1 head size is 107 by 80 nm and the tail length is 117 nm. A distinctive feature of phage Lw1 is its ability to spontaneously produce aberrant particles with abnormally elongated capsids (140 to 430 nm in length) at a higher frequency than in T4.

The virion DNA was obtained using the SDS-phenol extraction method. Sequencing was performed using the 454 pyrosequencing approach. The phage genome contig was assembled with Newbler version 2.5.3, which also confirmed its circular genetic map. Unconventionally, annotation of the Lw1 genome starts from the rIIB gene because of the nucleotide overlap between the rIIB and rIIA genes. The latter gene is the traditional start in T4-like phages, but the overlap prevents the genome from being displayed as a liner DNA molecule if started from rIIA.

The Lw1 virion contains a linear double-stranded DNA (dsDNA) molecule of 176,227 bp with circular permutation. There are many Dam and Dcm sites that are completely or partially methylated. The G+C content is 43.5% and the genome contains 274 predicted genes: 273 protein-coding open reading frames (ORFs) and 1 Met-tRNA gene. One hundred fifty ORFs (55%) have unknown functions. The gene density is 1.56 genes/kb and the coding density is 95.5%. The average gene size is 618 nucleotides, and the average protein size is 205 amino acids (aa). The closest relative phages, RB43, GAP161, RB16, KP27, and KP15, share with Lw1 91.2% (249/273 ORFs), 90.5% (247/273 ORFs), 89.7% (245/273 ORFs), 86.1% (235/273 ORFs), and 83.2% (227/273 ORFs) of homologs, respectively. Thus, phage Lw1 belongs to the RB43-group of pseudo T-even bacteriophages (7). Finally, although the Lw1 genome does contain one T4-like phage endonuclease of the MobE family, there is a lack of AP2-domain-containing endonucleases, and the relative paucity of mobile elements indicates either recent loss events or the presence of limited sites for viable insertion in the genome compared to some other RB43-like phages.

### Nucleotide sequence accession numbers.

This genome sequence has been deposited in GenBank with the accession no. KC801932. Described in this paper is the second version, accession no. KC801932.2.

## References

[1] Petrov VM Nolan JM Bertrand C Levy D Desplats C Krisch HM Karam JD 2006 Plasticity of the gene functions for DNA replication in the T4-like phages. J. Mol. Biol. 361:46–68 16828113 10.1016/j.jmb.2006.05.071

[2] Nolan JM Petrov V Bertrand C Krisch HM Karam JD 2006 Genetic diversity among five T4-like bacteriophages. Virol. J. 3:30. 10.1186/1743-422X-3-30 16716236 PMC1524935

[3] Kęsik-Szeloch A Drulis-Kawa Z Weber-Dąbrowska B Kassner J Majkowska-Skrobek G Augustyniak D Lusiak-Szelachowska M Zaczek M Górski A Kropinski AM 2013 Characterising the biology of novel lytic bacteriophages infecting multidrug resistant *Klebsiella pneumoniae*. Virol. J. 10:100. 10.1186/1743-422X-10-100 23537199 PMC3620542

[4] Abbasifar R Kropinski AM Sabour PM Ackermann HW Lingohr EJ Griffiths MW 2012 Complete genome sequence of *Cronobacter sakazakii* bacteriophage vB_CsaM_GAP161. J. Virol. 86:13806–13807 23166229 10.1128/JVI.02546-12 PMC3503131

[5] Magnani E Sjölander K Hake S 2004 From endonucleases to transcription factors: evolution of the AP2 DNA binding domain in plants. Plant Cell 16:2265–2277 15319480 10.1105/tpc.104.023135 PMC520932

[6] Ackermann HW Krisch HM 1997 A catalogue of T4-type bacteriophages. Arch. Virol. 142:2329–2345 9672598 10.1007/s007050050246

[7] Monod C Repoila F Kutateladze M Tétart F Krisch HM 1997 The genome of the pseudo T-even bacteriophages, a diverse group that resembles T4. J. Mol. Biol. 267:237–249 9096222 10.1006/jmbi.1996.0867

